# Supplementary material for: Chromothripsis during telomere crisis is independent of NHEJ, and consistent with a replicative origin
Source: Genome Res. 2019 May;29(5):737–49. doi: 10.1101/gr.240705.118 (PMC6499312; doi:10.1101/gr.240705.118)
Supplement: Supplemental Material [file supp_gr.240705.118_Supplemental_file_1.zip › contigs/annotated_contigs/DB89/contig.3.DB89_length_524_mean_cov_6.76908396947.docx]

**DB89_length_524_mean_cov_6.76908396947**

```
GTGGTAACACTTTTACTGATGGCAGCTTTGGCCAGTGGATGGGGAGTGCACATGTCACTCACATCTCAATCCCAGCAGCACTTTTGTCT
 >chr1:104359664-104359980 - E=1e-178
CAGTTCTGGGTGTGATAGCCTACAGTTGCTCACTCCTAAATCCTAGGGTCAGCAGCCAGCTCTTCCCTCTTGTTTTCTCAGCAGCCAAC

ATGGCAGAATCCAGCTCTGCTCCTGTCCCTCAGCCCCAGCACTGCAGAGCTCCAGGATAGCATGCAGTCTGCTCTGTTGGGGATGGGGC

TCTATGATGGTGTCTTGCTCTCTCTCTCTCTCTCTCTCTCTCTCTCTC|T|ATATATATATATAGAGTATCTATATATATATATAT|AG
                                                 >chr13:21581746-21581785 + E=8e-06 p=
T|ATCTGACATGCAATTTTTTTGCTAGATTTGCAAATAAATTGTGACCTGTTTACCTAATAGAAGTAATCATCACAATACTGACAACGG
5e-02  >chr1:104358407-104358580 - E=2e-92
TGAGGGAATTTTAGTTAACTAACAAAATGAGCCAGGCATCTTGCAAAAATATATATGTGCTTGACAAATTTTAATCATTTTCA
```
